# Root Transporters Shape Rhizosphere Microbiomes to Enhance Nitrogen Acquisition Efficiency in Plants

**DOI:** 10.3390/microorganisms14081609

**Published:** 2026-07-23

**Authors:** Izhar Ali, Xia Xu

**Affiliations:** 1 Zhejiang Key Laboratory of Carbon Sequestration and Emission Reduction in Agriculture and Forestry, Zhejiang A&F University, Hangzhou 311300, China; izharali48@gmail.com; 2 College of Environment and Resources, College of Carbon Neutrality, Zhejiang A&F University, Hangzhou 311300, China

**Keywords:** nitrogen use efficiency, rhizosphere microbiome, nitrogen cycling, amino acid transporters, plant–microbe interactions

## Abstract

Root nitrogen acquisition is a central belowground process that determines how efficiently plants capture nitrogen from the rhizosphere and influences fertilizer demand and environmental nitrogen losses. Root N transporters, including nitrate, ammonium, amino acid, and peptide transporters, provide the molecular basis for inorganic and organic N uptake. However, root N acquisition is not determined solely by plant transport systems but is also shaped by rhizosphere microbial communities that regulate N mobilization, transformation, and availability. In addition to bacteria and archaea, saprotrophic fungi and mycorrhizal associations contribute to organic matter decomposition, N mineralization, and symbiotic N transfer. Mechanistically, transporter activity may alter rhizosphere N gradients and substrate availability, while root exudates and microbial metabolites can influence microbial recruitment, root physiology, and transporter expression. Evidence from rice *NRT1.1B* and *OsLHT1* suggests that specific N transporter genes can influence rhizosphere microbiome assembly, although direct genetic evidence remains limited to a small number of transporter systems and crop contexts. This review synthesizes current knowledge on root N transporter diversity, rhizosphere microbial N cycling, organic N availability, and transporter–microbiome feedbacks in root-level N acquisition. By integrating plant physiology, soil microbiology, and rhizosphere ecology, this review proposes a conceptual framework in which root N transporters and microbial communities act as interconnected components of belowground N acquisition. Future integration of transporter-informed breeding, microbiome management, and fertilization strategies may improve root N capture while reducing reliance on synthetic N inputs.

## 1. Introduction

Nitrogen (N) is one of the most important macronutrients for plant growth and productivity, forming a fundamental component of amino acids, proteins, nucleic acids, and chlorophyll [1]. Despite its abundance in the atmosphere, bioavailable N in soils is often limited, making N availability a primary constraint on agricultural productivity worldwide [2,3]. To overcome this limitation, modern agriculture has relied heavily on synthetic N fertilizers, which have significantly enhanced crop yields over the past several decades. However, nitrogen recovery efficiency in agricultural systems remains low, as a substantial proportion of applied nitrogen is lost through leaching, volatilization, and microbial transformations, thereby contributing to environmental problems including eutrophication, groundwater contamination, and greenhouse gas emissions [4,5].

Traditionally, improving plant nitrogen acquisition has been approached through plant agronomic, physiological, and genetic strategies, particularly by optimizing N fertilization and enhancing root uptake of inorganic N forms such as nitrate and ammonium [6,7,8]. This has included extensive research on nitrate transporters (NRT families), ammonium transporters (AMTs), and associated regulated pathways involved in root N uptake. While these studies have provided important insights into the molecular basis of nitrogen uptake, they have often considered plants as largely autonomous systems that directly acquire N from soil [4,9].

However, increasing evidence challenges this simplified view by demonstrating that a significant proportion of soil N exists in organic forms, including amino acids and peptides, particularly in natural and low-input agricultural systems [2,10]. The availability of these organic N pools is not determined solely by plant transport systems but also depends strongly on microbial activity in soil. Soil microorganisms degrade organic matter and transform complicated nitrogenous chemicals into plant-accessible forms through enzymatic activities like proteolysis and mineralization [11,12]. These transformations are mediated by diverse microbial groups, including bacteria, archaea, saprotrophic fungi, and mycorrhizal associations, which collectively contribute to organic matter decomposition, N mineralization, and symbiotic N transfer [13,14,15]. As a result, N availability in the rhizosphere is increasingly understood as a microbially mediated process rather than a merely physicochemical phenomenon.

Additionally, the rhizosphere microbiome, a complex and dynamic microbial community shaped by root exudates and plant developmental processes, has emerged as a critical regulator of plant nutrient acquisition and stress tolerance [12,13,14,15,16]. Plants influence their associated microbial communities by releasing carbon-rich substances, signaling molecules, and secondary metabolites, which promote nutrient mobilization and uptake. This bidirectional relationship creates a functional interface between plant roots and soil microbial populations, where nutrient cycling is regulated by both plant and microbial partners [12,17].

Recent studies further suggest that plant genetic variation can influence the structure and function of rhizosphere microbial communities, indicating that microbiome assembly is at least partially under host genetic control [12,16]. In particular, genes involved in nutrient transport and root exudation have been implicated in shaping microbial community composition, thereby linking plants to belowground ecosystem processes. For example, natural variation in the rice amino acid transporter *OsLHT1* between japonica and indica subspecies is associated with differential recruitment of rhizosphere bacteria that enhance organic N acquisition [12]. This emerging concept challenges the traditional separation between plant physiology and soil microbiology and instead supports the view that plant nutrient acquisition is a systems-level property emerging from plant–microbe interactions [5,12].

In this review, nitrogen acquisition refers specifically to the belowground processes through which plant roots capture nitrogen from the rhizosphere, including transporter-mediated uptake of nitrate, ammonium, amino acids, and peptides, as well as microbially mediated mobilization of N from organic and inorganic soil pools. Accordingly, the review focuses on root-level nitrogen capture rather than whole-plant nitrogen utilization.

Despite recent advances, several fundamental questions remain unresolved. First, the mechanisms by which plant N transporters influence rhizosphere microbiome assembly remains poorly understood. Second, the extent to which microbial communities enhance transporter-mediated N acquisition, particularly organic N uptake, is not fully established. Third, most studies have examined plant N transport systems and microbial N cycling independently, with limited integration of both systems into a unified framework. Therefore, an integrated perspective is required to clarify how root N transporters and rhizosphere microbial communities interact to regulate N acquisition. The central premise of this review is that root N transporters contribute to N acquisition not only by mediating uptake of nitrate, ammonium, amino acids, and peptides, but also by shaping rhizosphere nutrient gradients and microbial functions involved in N mobilization. In turn, rhizosphere microorganisms may feed back to influence N availability and transport activity, forming reciprocal plant–microbe interactions that regulate root-level N capture.

Therefore, this review summarizes current knowledge on major root N transport systems involved in inorganic and organic N uptake, rhizosphere microbial N cycling, and transporter–microbiome interactions that contribute to plant N acquisition. Emphasis is placed on how transporters involved in nitrate, ammonium, amino acid, and peptide uptake may interact with microbial processes that regulate N mobilization in the rhizosphere. By integrating evidence from plant physiology, soil microbiology, and rhizosphere ecology, this review proposes a conceptual framework in which root N transporters and microbial communities function as interconnected components of belowground N acquisition.

## 2. Root Nitrogen Transport Systems Involved in Nitrogen Acquisition

Root nitrogen acquisition relies on specialized transport proteins that mediate the uptake of inorganic and organic N forms from the rhizosphere under fluctuating soil conditions [2,3]. These transport systems include nitrate transporters (NRTs), ammonium transporters (AMTs), amino acid transporters, and peptide transporters, which collectively determine the capacity of roots to capture N from soil N pools. Although some N transporters also participate in signaling and internal N distribution, this section focuses primarily on their roles in root N acquisition [8,18].

### 2.1. Molecular Diversity of Root N Transporters

Nitrate uptake is primarily mediated by members of the NPF (formerly *NRT1*) and *NRT2* transporter families, which operate across variable soil nitrate concentrations. *NPF*/*NRT1* transporters generally participate in low-affinity nitrate uptake and nitrate distribution, whereas NRT2 transporters are mainly involved in high-affinity nitrate uptake under nitrate-limiting conditions [5,6,19]. NPF6.3 (*CHL1/NRT1.1*) is a well-characterized dual-affinity nitrate transporter and nitrate sensor that integrates external nitrate availability with root development and transcriptional responses [17,20]. Its affinity state is regulated by phosphorylation of threonine 101, enabling plants to dynamically adjust nitrate uptake according to external nitrate supply [21]. Several *NRT2* family members, particularly *NRT2.1*, *NRT2.2*, *NRT2.4* and *NRT2.5*, contribute to nitrate uptake under nitrogen-limiting conditions, with *NRT2.1* alone accounting for up to 75% of root nitrate influx [22,23]. Most *NRT2* proteins require the partner protein NAR2.1 to form functional complexes [24].

Ammonium uptake is mainly mediated by AMT1 and AMT2 family proteins. AMT1-type transporters are generally associated with high-affinity ammonium uptake at the root-soil interface, whereas AMT2-type members contribute to ammonium transport under specific tissue and environmental contexts [8,25]. Because ammonium is both an essential N source and potentially toxic at high concentrations, plants employ tight regulatory mechanisms, including phosphorylation-based inhibition and feedback regulation by glutamine and other metabolites [26,27]. The balance between nitrate and ammonium uptake is highly context-dependent, influenced by soil pH, oxygen availability, microbial activity, and plant developmental stage [28,29].

In addition to inorganic N, roots can acquire organic nitrogen forms, particularly amino acids and small peptides released during microbial decomposition of soil organic matter [2,10]. Amino acid transport is mediated by several members of transporter families, including amino acid permeases (AAPs), lysine-histidine transporters (LHTs), proline transporters (ProTs), GABA transporters (GATs), cationic amino acid transporters, and UMAMIT-type transporters, as well as the PTR/POT family for di- and tripeptides [9,30,31,32]. These families differ in substrate specificity, tissue localization, and transport direction, allowing plants to acquire and redistribute diverse organic N compounds [31,33]. Among these, LHTs and AAPs are particularly relevant to organic N acquisition because amino acids can be directly absorbed by roots and also represent major mobile forms of organic N within plants [31,33]. LHT1 has been widely studied as a high-affinity amino acid transporter in *Arabidopsis* and rice [34,35]. In rice, natural variation in *OsLHT1* distinguishes japonica (*OsLHT1a*) from indica (*OsLHT1b*), with the *OsLHT1a* haplotype associated with higher aspartate uptake and recruitment of rhizosphere microbiota that promote soil organic N acquisition [10,12].

Peptide transporters also contribute to organic N acquisition by mediating the uptake and movement of small peptides. For example, *AtPTR1* and *AtPTR2* transport di- and tripeptides via proton-coupled mechanisms [36,37]. Members of the *NRT1/PTR/POT/NPF* transporter superfamily can transport nitrate and/or peptides, indicating functional overlap between inorganic and organic N transport systems [38]. This function is particularly relevant in the rhizosphere, where microbial proteolysis releases peptides and amino acids before complete mineralization to ammonium or nitrate. Therefore, amino acid and peptide transporters provide an important molecular link between microbial organic N mobilization and root N acquisition [18].

### 2.2. Functional Diversity of Root N Transporters in N Acquisition

The functional diversity of root N transporters extends beyond substrate uptake. These proteins can participate in nutrient sensing, uptake regulation, pH homeostasis, coordination with plant carbon and hormonal status, and adjustment of root responses to changing rhizosphere N availability [8,39,40,41]. For example, *NPF6.3/NRT1.1* functions both as a nitrate transporter and nitrate sensor, linking external nitrate availability with calcium-dependent signaling, NLP transcription factor activation, and nitrate-responsive gene expression [42,43]. Similarly, AMT-mediated ammonium uptake is tightly regulated by external ammonium supply and internal N status, allowing plants to balance ammonium acquisition with toxicity avoidance and cellular pH regulation [8,44,45]. Root N transporters can also modify the chemical environment at the soil–root surface. High-affinity uptake systems for nitrate, ammonium, amino acids, and peptides may reduce the local availability of these N forms near active root surfaces, thereby generating localized nutrient depletion zones in the rhizosphere [46,47]. Such rhizosphere gradients may influence microbial competition, N transformation, and the enrichment of microorganisms capable of mobilizing or releasing specific N substrates [46,48]. Therefore, transporter activity may indirectly shape rhizosphere microbial assembly by modifying N availability in the immediate root environment.

Emerging evidence further indicates that root N transporter function can be integrated with microbial activity in the rhizosphere [16,17]. For example, in rice, *NRT1.1B*, which encodes a nitrate transporter and sensor, is associated with root microbiota composition and microbiota functions related to N transformation under field conditions [11]. Nitrate signaling via *NPF6.3/NRT1.1* influences root developmental responses and auxin transport, which may indirectly affect microbial colonization [49]. Moreover, natural variation in OsLHT1 was shown to influence rhizosphere microbiota assembly and enhance soil organic N acquisition by promoting amino acid production and root amino acid uptake [10].

**Figure 1:**
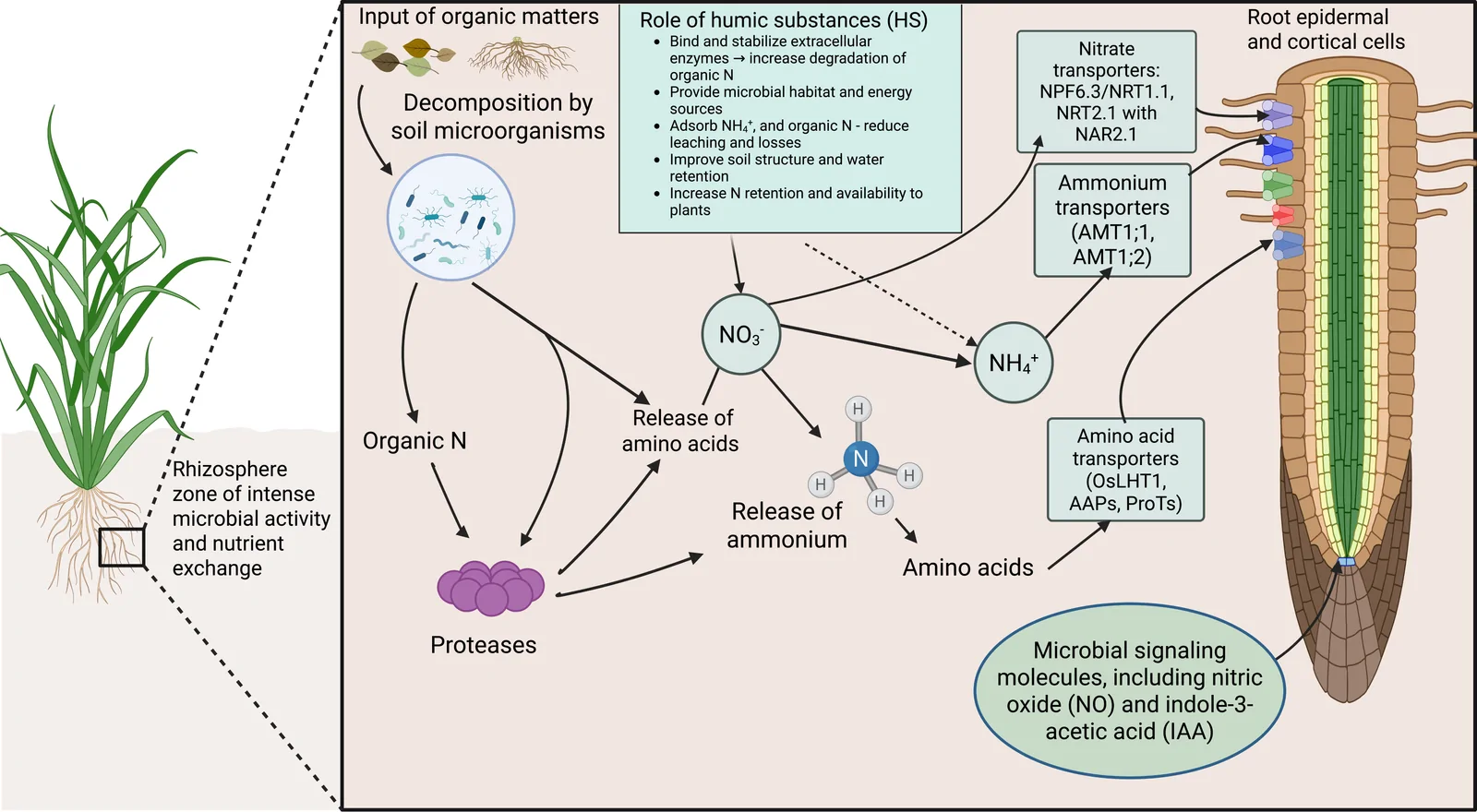
Schematic illustration of rhizosphere nitrogen (N) cycling and root N uptake mediated by soil microorganisms, humic substances, and plant transport systems. Soil microorganisms decompose organic matter, releasing amino acids, ammonium (NH_4_^+^), and nitrate (NO_3_^−^,), while humic substances enhance organic N decomposition, nutrient retention, and N availability. Root epidermal and cortical cells absorb NO_3_^−^, NH_4_^+^, and amino acids through specific nitrate, ammonium, and amino acid transporters. Microbial signaling molecules, including nitric oxide (NO) and indole-3-acetic acid (IAA), further regulate root N uptake and plant–microbiome interactions. Created using BioRender (Toronto, ON, Canada; app.biorender.com).

Despite significant advances, several limitations remain. Most studies have focused on individual transporter families in isolation, without fully considering their interactions within the broader root nitrogen acquisition network. Direct evidence linking transporter activity with rhizosphere microbiome assembly remains limited and is currently concentrated in a small number of cases, particularly *NRT1.1B* and *OsLHT1* in rice. The contributions of AMTs, AAPs, PTR/POT transporters, and other organic N transporters to microbiome-mediated N acquisition remain poorly resolved, especially under field conditions [10,11]. Addressing these gaps requires a systems-level perspective that integrates plant transporter biology, soil microbial ecology, and rhizosphere N biogeochemistry. The coordinated interactions between microbial nitrogen transformations and plant nitrogen transport systems are summarized in Figure 1.

## 3. Rhizosphere Microbial Nitrogen Cycling and Organic Nitrogen Availability

### 3.1. Rhizosphere Microbial N Cycling

Soil microbial communities are primary regulators of N transformation in terrestrial ecosystems, controlling the conversion of organic and inorganic N pools into plant-available forms through a complex network of biochemical processes [2,3]. These processes include biological N fixation, organic matter decomposition, proteolysis, ammonification, nitrification, denitrification, and dissimilatory nitrate reduction to ammonium, all of which influence the size, chemical form, and persistence of available N pools in the rhizosphere [5,13,34,50]. Functional groups involved in these processes include N-fixing bacteria, ammonia-oxidizing bacteria, ammonia-oxidizing archaea, denitrifying bacteria, and DNRA-associated microbial guilds, whose abundance and activity vary with soil type, pH, N source, and fertilization regime [13,50].

A key process in microbial N cycling is the enzymatic breakdown of complex organic substrates such as proteins, peptides, and plant residues into smaller N-containing molecules [2,10]. Extracellular enzymes, including proteases, peptidases, and deaminases, depolymerize organic N into peptides, amino acids, ammonium, and other low-molecular-weight N compounds that can be further transformed by microorganisms or taken up by plant roots [2,5,18,51]. The efficiency of these decomposition and mineralization processes strongly influences the availability of organic and inorganic N forms in the rhizosphere.

Microbial communities involved in N cycling are taxonomically and functionally diverse. Proteolytic and mineralizing bacteria, including members of Bacillus, Pseudomonas, Streptomyces, and other decomposer groups, contribute to protein degradation, amino acid release, and ammonium production [12]. Archaea, particularly ammonia-oxidizing archaea, contribute to nitrification by oxidizing ammonium-derived substrates under specific soil conditions [13]. Fungal decomposers also play important roles in organic matter turnover. Saprotrophic fungi contribute to the breakdown of plant residues and complex organic materials, whereas mycorrhizal fungi provide additional pathways for N mobilization and plant acquisition [2,14,15,52]. The inclusion of fungal pathways is essential for a complete understanding of rhizosphere N cycling. Saprotrophic fungi are important decomposers of organic matter and can influence the release of mineral N from organic substrates [15]. Arbuscular mycorrhizal fungi can facilitate host-plant N uptake and participate in N assimilation and translocation within the symbiosis [14]. Ectomycorrhizal fungi are particularly important in forest and organic-rich soils, where they can mobilize N from organic or mineral-associated forms and transfer part of this N to host plants [52]. These fungal and mycorrhizal pathways expand the rhizosphere microbiome beyond bacteria and provide an important biological route linking soil organic N mobilization with plant N acquisition.

In addition to mineralization, microbial immobilization strongly regulates N availability by incorporating inorganic and organic N into microbial biomass. This process can reduce immediate N availability to plants, but microbial turnover subsequently contributes to N recycling and long-term soil fertility [51]. Therefore, rhizosphere N availability reflects a dynamic balance between microbial mineralization, immobilization, nitrification, denitrification, symbiotic transfer, and plant uptake.

Plant-derived inputs further regulate microbial N cycling. Root exudates provide carbon substrates and signaling molecules that shape microbial community composition and activity, while microbial N transformations modify the availability of nitrate, ammonium, amino acids, and peptides near the root surface [50,51]. Thus, rhizosphere N cycling should be viewed as a coupled plant–microbe process in which roots influence microbial activity through exudation and nutrient uptake, while microorganisms regulate the N forms available for transporter-mediated acquisition.

### 3.2. Organic N Availability and Microbial Mediation of Root N Acquistion

Organic N represents a major component of soil N pools, particularly in natural, organic-rich and low-input agricultural systems. Its availability to plants depends on microbial depolymerization, mineralization, immobilization, and competition between roots and microorganisms [4,51,53]. Amino acids and small peptides are especially relevant to root N acquisition because they are direct products of microbial proteolysis and can be absorbed by roots through amino acid and peptide transport systems [18]. Recent evidence from rice and wheat confirms that crop roots can acquire exogenous N from amino acids and oligopeptides, although uptake capacity differs between speciews and along root regions [18].

The availability of amino acids and peptides in the rhizosphere is highly dynamic because microbial production, microbial uptake, mineralization, and root absorption occur simultaneously [51,53]. Microorganisms often compete strongly with plants for dissoleved organic N because of their rapid uptake capacity and close contact with soil organic substrates [4,51]. However, microbial activity can also enhance plant N acquisition by decomposing organic matter, releasing amino acids and ammonium, stimulating rhizosphere priming, and forming symbiotic associations that transfer N to host plants [4,14,51,53]. Therefore, plant–microbe interactions for organic N should be viewed as a balance between competition and cooperation rather than as a strictly antagonistic process.

Amino acids provide an important interface between microbial metabolism and transporter-mediated N acquisition. They can be released from soil organic matter through microbial proteolysis, consumed by microorganisms, mineralized to ammonium, or directly absorbed by roots through amino acid transporters such as LHTs and AAPs [18,53]. Small peptides provide an additional organic N pathway because microbial proteolysis generates oligopeptides before complete mineralization, and crop roots can absorb peptide-derived N [18]. These processes provide a mechanistic link between microbial organic N mobilization and the amino acid and peptide transporter families discussed.

Mycorrhizal associations provide another route through which microbial processes contribute to root N acquisition. Arbuscular mycorrhizal fungi can facilitate plant N uptake and interact with host N assimilation and translocation pathways [14,54]. Ectomycorrhizal fungi can mobilize N from organic matter and mineral-associated pools, particularly in systems where organic N dominates soil N availability [52,55]. Fungal functional guilds differ in their effects on N cycling; ectomycorrhizal, arbuscular mycorrhizal, ericoid mycorrhizal, and saprotrophic fungi have been associated with different patterns of soil N mineralization and ammonification [14,15,52]. These findings indicate that fungal and mycorrhizal pathways should be integrated into models of rhizosphere-mediated N acquisition.

Plant-derived inputs further regulate organic N availability. Root exudates provide carbon substrates and signaling molecules that shape microbial activity, while exudate composition and C stoichiometry can influence microbial N mining, mineralization, and nitrification in the rhizosphere [12,16,56]. Thus, root exudation, microbial organic N mobilization and transporter-mediated uptake operate as connected processes rather than independent components of soil N cycling [12,53,56].

For example, microbial metabolites and signaling compounds can influence root architecture, nutrient transporter expression, and physiological responses in plants, thereby directly affecting N uptake efficiency [3,12]. This suggests that N cycling in soils is governed by a coupled plant–microbe regulatory system rather than independent biological processes.

Despite these advances, several key gaps remain. First, the spatial and temporal dynamics of amino acid and peptide production, microbial immobilization, and root uptake remain difficult to quantify under field conditions [2,4,10,18,51]. Second, the relative contributions of different microbial taxa to N mineralization and organic N transfer vary across soil types, plant species, and management regimes [13,15,50,52]. Third, microbial N cycling is still often studied separately from plant transporter activity, limiting our ability to explain how microbial N mobilization is coupled with transporter-mediated N acquisition [12,50,53]. Addressing these gaps requires integrated approaches that combine microbial functional profiling, plant physiology, and soil biogeochemistry.

Overall, rhizosphere microbial N cycling provides the biochemical foundation for root N acquisition system by converting organic and inorganic soil N pools into plant-accessible forms. The transformation of organic N into plant-available forms is not a passive process but is actively regulated by microbial community structure, enzymatic activity, and plant-derived inputs. Understanding these processes is essential for developing strategies to improve N use efficiency through targeted manipulation of plant–microbe interactions.

## 4. Rhizosphere Microbiome and Nutrient Exchange Between Plants and Rhizosphere Microbiome

The rhizosphere microbiome functions as a plant-shaped microbial interface where root-derived carbon inputs, microbial nutrient transformations, and reciprocal signaling regulate nutrient availability and root nutrient acquisition [2,16,57]. Recent reviews emphasize that microbiome-facilitated nutrient acquisition involves plant signaling, microbial sensing, and nutrient exchange at the soil–root interface [57]. Unlike bulk soil, the rhizosphere is continuously modified by root exudates—a complex mixture of carbohydrates, organic acids, amino acids, and secondary metabolites—that selectively stimulate or inhibit specific microbial taxa [12]. This plant-driven selection establishes a bidirectional signaling network in which microbes influence root physiology and transporter activity, while plants modulate microbial community structure and function.

The composition of root exudates is tightly controlled by plant nitrogen status and transporter activity; under nitrogen limitation, plants increase the release of carbon-rich compounds and specific amino acids, which serve as chemo-attractants and carbon sources for proteolytic and mineralizing bacteria [3]. In rice, natural variation in *OsLHT1* provides one example of transporter-associated rhizosphere microbiome assembly, in which the *OsLHT1a* allele is linked with enhanced amino acid acquisition and recruitment of a distinct rhizosphere microbiome [10]. Mechanistically, selective enrichment is mediated by bacterial chemotaxis receptors and quorum-sensing systems that recognize specific root exudate components [49]. Once established, these microbes produce extracellular enzymes—proteases, peptidases, and deaminases—that hydrolyze soil organic nitrogen into amino acids and ammonium, directly feeding the plant’s transport systems [2,9].

Beyond nutrient provisioning, rhizosphere bacteria actively regulate plant nitrogen transporter expression through diffusible signals. Ma et al. (2026) isolated four bacterial strains (AM16, AM17, AM26, AM28) from *OsLHT1a* rhizosphere that significantly upregulated *OsLHT1* transcript levels in rice roots (relative expression > 2.0) without increasing soil amino acid concentrations [10]. It suggested the production of transporter-activating signals. Although the molecular identity of these signals remains unknown, candidates include volatile organic compounds, lipopeptides, and N-acyl homoserine lactones, which in other systems modulate nutrient transporter gene expression [49]. This microbial regulation creates a positive feedback loop: efficient amino acid uptake via *OsLHT1* reduces rhizosphere amino acid concentrations, which may further stimulate microbial signal release, reinforcing the interaction. In addition to bacterial processes, fungal and mycorrhizal pathways should be included in this nutrient-exchange framework because arbuscular mycorrhizal fungi contribute to plant N uptake and translocation, while ectomycorrhizal fungi can mobilize N from organic and mineral-associated pools [14,52].

Moreover, microbial metabolites directly influence root architecture and transporter kinetics. For instance, microbial auxin (IAA) production promotes lateral root branching, expanding the rhizosphere interface and increasing the density of transporter-expressing root cells [3]. Similarly, microbial-derived nitric oxide (NO), produced via nitrate reductase activity, modulates the primary nitrate response (PNR) by S-nitrosylation of *NLP7*, a master transcription factor that regulates *NRT2.1* and other nitrate transporter genes [42]. This represents a direct post-translational control of plant nitrogen signaling by microbial activity. Conversely, plant nitrogen transporter activity shapes the chemical environment of the rhizosphere. High-affinity uptake systems (e.g., *NRT2.1*, *OsLHT1*) rapidly deplete inorganic and organic nitrogen from the soil solution, creating a nitrogen-limited microzone that favors nitrogen-scavenging microbes [5,10]. This selective pressure enriches microbial taxa with efficient proteolytic and amino acid transport systems, thereby co-evolving plant and microbial nitrogen acquisition traits.

Functional redundancy is common in soil microbiomes, yet specific microbial functions are enriched by particular plant genotypes. Ma et al. (2026) [10] constructed a synthetic microbiota (SynM) comprising four amino-acid-producing strains (AM1, AM2, AM6, AM9) and four *OsLHT1*-upregulating strains (AM16, AM17, AM26, AM28). This SynM synergistically enhanced organic nitrogen uptake in rice, but only in *OsLHT1a* haplotypes grown in organic-fertilized soils, demonstrating genotype- and environment-dependent specificity. The mechanistic basis lies in the requirement of functional *OsLHT1* for both amino acid import and, indirectly, for maintaining the chemical gradients that sustain the enriched microbial community.

Despite these advances, key mechanisms remain unresolved: the molecular identity of microbial signals that regulate plant transporter expression, the root receptors and signaling pathways that perceive these cues, and the temporal dynamics of exudate-mediated recruitment versus transporter-mediated feedback under field conditions [10]. Overall, the rhizosphere functions as a co-regulated system where plant transporter genes and microbial functional traits are linked through reciprocal signaling and nutrient exchange. This bidirectional coupling creates a self-reinforcing nitrogen acquisition module that can be harnessed to improve nitrogen use efficiency through genotype-matched microbiome engineering.

## 5. Gene–Microbiome Coupling in Root Nitrogen Acquisition

Nitrogen acquisition in plants cannot be fully explained by transporter activity or microbial nitrogen cycling alone; instead, it emerges from a coordinated interaction between plant genetic regulation and rhizosphere microbial community assembly—a concept increasingly referred to as gene–microbiome coupling [12,16]. This framework proposes that plant genotypes actively shape microbial communities through root exudation and nutrient uptake, and these microbes in turn feedback to influence nitrogen availability and transporter expression, creating an integrated, co-regulated system rather than two independent biological processes [2,3]. Plant genetic variation has been shown to significantly influence rhizosphere microbial composition, particularly through genes involved in nutrient transport. In rice, natural variation in the amino acid transporter *OsLHT1* distinguishes japonica (*OsLHT1a*) from indica (*OsLHT1b*) haplotypes. The *OsLHT1a* haplotype, which confers higher affinity for acidic amino acids and amides, is associated with a distinct rhizosphere bacterial community enriched in taxa involved in amino acid production from organic matter decomposition [10]. This indicates that transporter genes act not only in nutrient uptake but also as indirect ecological engineers of the rhizosphere microbiome, likely by modulating the concentration and composition of amino acids and other nitrogenous compounds in the rhizosphere soil solution. Depletion of specific amino acids by high-affinity uptake creates a selective environment that favors microbes capable of producing those same compounds, establishing a mutualistic reinforcement loop [10,35].

Mechanistically, gene–microbiome coupling operates through several interconnected pathways. First, root exudation patterns are directly influenced by transporter activity and plant nitrogen status. Under nitrogen limitation, plants increase the release of carbon-rich compounds and amino acids, which stimulate microbial proteolytic activity and nitrogen mineralization [3]. Second, microbial metabolites and signaling molecules can feedback to regulate plant transporter gene expression. Ma et al. [10] demonstrated that specific bacterial strains isolated from *OsLHT1a* rhizosphere upregulate *OsLHT1* transcript levels in rice roots, an effect that requires a functional *OsLHT1* gene itself, suggesting a self-reinforcing transcriptional loop. Third, microbial-derived nitric oxide (NO) has been shown to modulate the primary nitrate response (PNR) by S-nitrosylation of the *NLP7* transcription factor, which directly regulates the expression of nitrate transporter genes such as *NRT2.1* [42]. This represents a direct post-translational link between microbial activity and plant nitrogen transport.

A particularly compelling example of gene–microbiome coupling comes from field studies across diverse rice cultivars. Zhang et al. [11] showed that the nitrate transporter gene *NRT1.1B* is associated with root microbiota composition and nitrogen use efficiency in rice. Similarly, Ma et al. [10] demonstrated that the *OsLHT1a* haplotype not only exhibits higher amino acid uptake but also enriches a synthetic microbiota (SynM) that enhances organic nitrogen acquisition. Importantly, this enrichment and the resulting growth promotion were heritable across generations only when the functional *OsLHT1* allele was present, and only under organic fertilization conditions. In contrast, the lht1/lht1 mutant failed to maintain SynM colonization and showed no response to microbial inoculation, providing direct genetic evidence that the transporter is essential for establishing and maintaining the beneficial plant–microbe partnership [10].

The concept of gene–microbiome coupling also extends to systemic signaling. Nitrogen transporters such as NPF6.3 (NRT1.1) have been shown to influence root system architecture by mediating auxin transport, thereby indirectly affecting microbial colonization patterns and nutrient uptake efficiency [3,49]. These observations suggest that gene–microbiome coupling is not limited to a single transporter but involves a network of nutrient and hormone signaling pathways that collectively determine how plants recruit and sustain beneficial microbial communities.

Current evidence suggests that transporter-microbiome coupling may operate through at least three non-exclusive pathways: transporter-driven changes in rhizosphere nutrient gradients, transporter-associated changes in root exudation or substrate availability, and microbial feedback regulation of transporter expression. However, direct genetic evidence remains limited. The strongest examples are *NRT1.1B*-associated root microbiota composition in rice and *OsLHT1*-mediated microbiome assembly linked with organic N acquisition [10,11].

**Figure 2:**
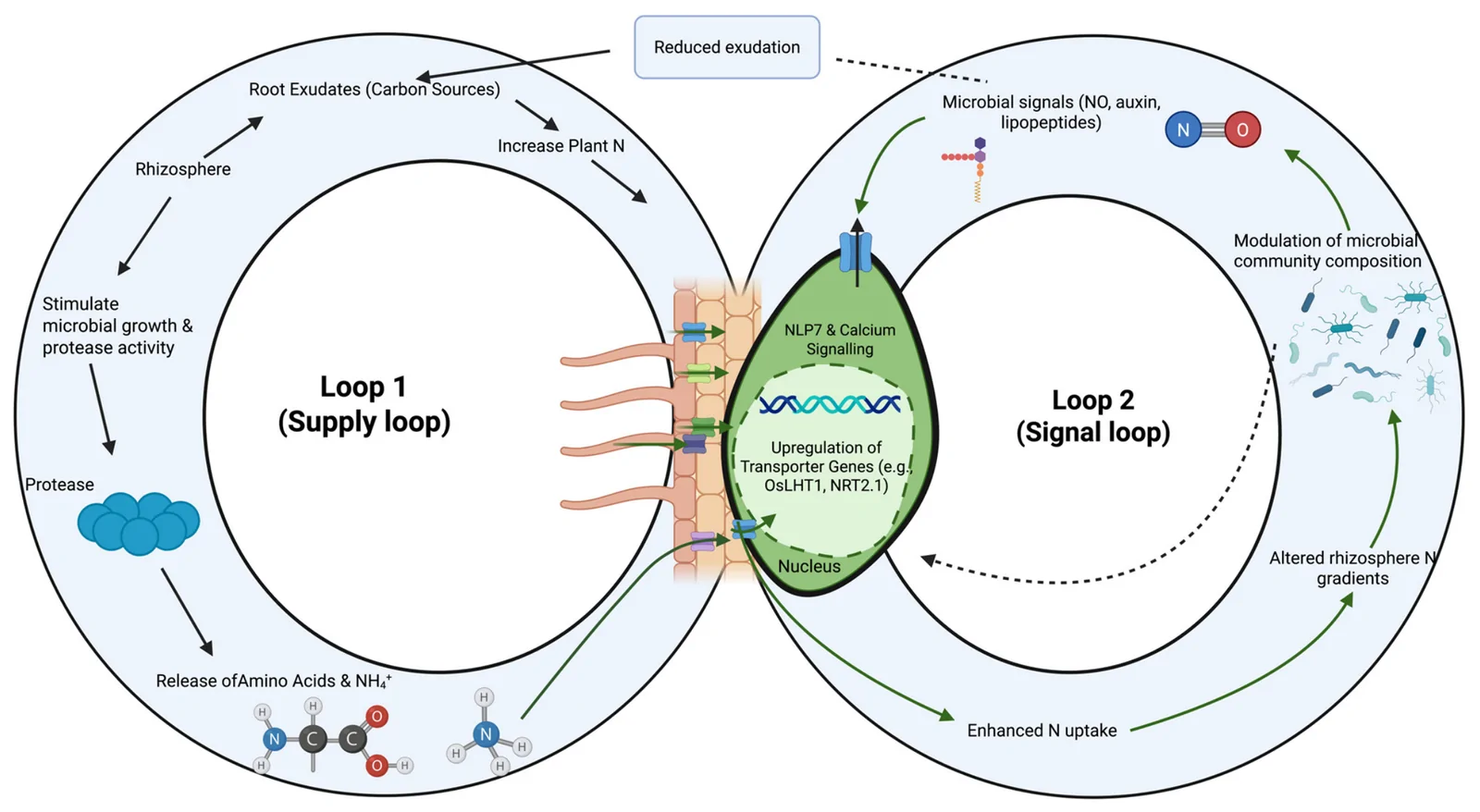
Bidirectional feedback loops linking root nitrogen transporters and rhizosphere microorganisms. Loop 1 (supply loop) illustrates how root exudates stimulate microbial growth and protease activity, promoting organic matter decomposition and the release of amino acids and ammonium (NH_4_^+^), which are absorbed by roots through specific transporters. Improved plant nitrogen status subsequently modifies root exudation, completing the nutrient feedback cycle. Loop 2 (signal loop) shows how microbial signaling molecules, including nitric oxide (NO), auxins, and lipopeptides, activate root signaling pathways (e.g., *NLP7* and calcium signaling), leading to the upregulation of nitrogen transporter genes (e.g., *OsLHT1* and *NRT2.1*). Enhanced nitrogen uptake alters rhizosphere nitrogen gradients and microbial community composition, reinforcing plant–microbe signaling and feedback regulation. Created using BioRender (Toronto, ON, Canada; app.biorender.com).

Despite these advances, several mechanistic gaps remain. The molecular identity of microbial signals that directly regulate plant transporter expression is still unknown, as are the root receptors that perceive these signals. Furthermore, the extent to which gene–microbiome coupling is conserved across different crop species and soil environments requires systematic investigation [2,10]. These findings suggest that root N acquisition can, in specific genetic and soil contexts, emerge from interaction between plant transporter activity and rhizosphere microbial function. However, broader generalization across transporter families, crop species, and field environments remain premature. This perspective opens new avenues for breeding crops that are not only efficient in nutrient uptake but also capable of recruiting and maintaining beneficial microbial consortia, thereby reducing dependence on synthetic fertilizers and enhancing agricultural sustainability. The bidirectional feedback interactions between root transporters and rhizosphere microorganisms are illustrated in Figure 2.

## 6. Feedback Regulation Among Roots, N Transporters, and Rhizosphere Microbial Communities

Nitrogen is regulated by feedback loops linking root physiological status, N transporter activity, and rhizosphere microbial community function [2,3]. Rather than operating as independent components, root transporters, and microorganisms form a coupled belowground system in which nutrient fluxes, microbial metabolism, and transporter expression influence one another [10].

### 6.1. Feedback Regulation Between Roots and N Transporters

Root N transporters are regulated by plant N status, carbon availability, and hormone-related signaling pathways. NPF6.3 (NRT1.1) functions not only as a nitrate transporter but also as a nitrate sensor that links external nitrate availability with root developmental responses, auxin transport, and nitrate-responsive gene expression [16,49,58]. Nitrate-triggered calcium signaling and NLP transcription factors are central components of the primary nitrate response, allowing plants to dictate nitrate uptake with transcriptional regulation and root adaptation [59,60]. Similarly, AMT-mediated ammonium uptake is regulated by external ammonium supply and internal N status to maintain ammonium acquisition while avoiding toxicity and pH imbalance [8]. These regulatory processes connect root physiological status with transporter activity and allow plants to adjust N acquisition under changing rhizosphere conditions.

### 6.2. Feedback Regulation Between Roots and Rhizosphere Microbial Communitiies

Root exudation acts as a central mediator through which plants regulate rhizosphere microbial communities. The quantity and composition of root exudates, including sugars, organic acids, and amino acids, are strongly influenced by plant developmental stage, nutrient status, and environmental conditions [3,61,62]. Under nitrogen limitation, the release of carbon-rich compounds can stimulate microbial growth, protease activity, and organic N mineralization, thereby increasing the release of amino acids and ammonium [2]. Once plant N status improves, shifts in exudation may reduce microbial stimulation and contribute to feedback regulation of rhizosphere N availability. In this way, roots shape microbial community structure and function by altering environment the chemical of the rhizosphere, while bacterial activity feeds back to influence the N forms available for root acquisition.

### 6.3. Feedback Regulation Between N Transporters and Rhizosphere Microbial Communitiies

Rhizosphere microorganisms can influence transporter-mediated N acquisition by producing metabolites and signaling molecules that regulate root physiology and transporter expression. For example, nitric oxide is required for the primary nitrate response and has been linked with S-nitrosation of NLP7, suggesting a route through which microbial or plant-derived NO may influence nitrate-responsive signaling [63]. In rice, specific rhizosphere bacteria associated with the OsLHT1a haplotype can upregulate *OsLHT1* expression, indicating that microbial signals may feed back to regulate transporter-mediated organic N acquisition [10].

Conversely, transporter activity can influence microbial community structure by modifying N availability near the root surface. High-affinity uptake systems for nitrate, ammonium, amino acids, and peptides may generate localized depletion zones that favor microorganisms capable of mobilizing or releasing the depleted N forms. In rice, *NRT1.1B* is associated with root microbiota composition and microbial functions related to N transformation under field conditions [11]. Natural variation in *OsLHT1* is also linked with rhizosphere microbiota assembly and enhanced soil organic N acquisition, particularly through microbial amino acid production and root amino acid uptake [10]. However, the extent to which similar transporter-driven microbial selection occurs for AMTs, AAPs, PTR/POT transporters, or other crop species remains unclear.

The *OsLHT1*-SynM system provides a useful example of transporter-microbiome feedback, but it should be interpreted cautiously. SynM-mediated enhancement of organic N acquisition depends on the functional *OsLHT1* allele and organic fertilization context, indicating that transporter-microbiome feedback is genotype- and environment-dependent rather than universally transferable [10].

Despite these insights, several mechanistic gaps remain. The molecular identity of microbial signals that directly regulate plant transporter expression is still unknown. Despite these insights, several mechanistic gaps remain. The molecular identity of microbial signals that regulate transporter expression is still poorly understood, and the plant receptors or signaling pathways that perceive these cues remain largely unresolved. The relative contribution of direct feedback, nutrient-mediated such as rhizosphere N depletion, versus indirect signal-mediated feedback, such as microbial metabolites or phytohormones, also requires quantitative testing. In addition, most evidence comes from a limited number of rice transporter examples, especially *NRT1.1B* and *OsLHT1*, whereas feedback roles of AMTs, AAPs, PTR/POT transporters, and mycorrhiza-associated N transport pathways remain underexplored [10,11].

Overall, feedback regulation among roots, N transporters, and rhizosphere microbial communities provides a useful framework for understanding belowground N acquisition. These interactions connect transporter-mediated uptake, microbial N mobilization, root exudation, and microbial signaling. However, broader validation across transporter families, microbial groups, crop species, and field environments is required before these feedback mechanisms can be generalized.

## 7. Agricultural Implications: Engineering Nitrogen Acquisition Through Plant–Microbiome Integration

The emerging understanding of root N acquisition as a coupled plant–microbiome process provides a conceptual foundation for improving plant N capture from the rhizosphere while reducing reliance on external N inputs. Microbiome-facilitated nutrient acquisition involves plant signaling, microbial sensing, nutrient mobilization, and nutrient exchange at the soil–root interface. Therefore, agricultural strategies should consider not only crop genotype and fertilizer management but also the capacity of roots to recruit and sustain functions that enhance rhizosphere N availability [57,64,65,66].

One of the most promising strategies is the targeted breeding of crop varieties with enhanced capacity to recruit beneficial rhizosphere microbiomes. Plant genotypes differ significantly in their ability to shape microbial community composition through root exudation patterns and transporter activity [2,10,67,68,69]. In rice, the japonica *OsLHT1a* haplotype not only exhibits higher amino acid uptake but also consistently enriches a rhizosphere bacterial community specialized in organic nitrogen decomposition and amino acid release [10]. This suggests that transporter-informed breeding may contribute to improved root N acquisition when combined with suitable soil and microbial management [67,68].

Synthetic microbial communities and microbial inoculants also offer potential tools for enhancing rhizosphere N mobilization. Microbial consortia that promote organic matter decomposition, amino acid production, ammonium release, or transporter expression may improve the coordination between microbial N supply and root N uptake. However, microbiome-based strategies are unlikely to function as universal inoculants. The *OsLHT1* case indicates that beneficial microbial effects can depend on host transporter genotype and organic fertilization context, highlighting the need to match crop genotype, microbial consortium, and soil management regime [10,69].

A key implication is that microbiome-based strategies are unlikely to function as universal inoculants. The *OsLHT1* case indicates that beneficial microbial effects can depend on host transporter genotype and organic fertilization context, highlighting the need to match crop genotype, microbial consortium, and soil management regime. This genotype-environment-microbe compatibility should be considered when designing transporter-informed microbiome strategies for root N acquisition [10].

The agricultural application of microbiome-based N acquisition strategies remains challenging. Synthetic microbial communities and microbial inoculants may perform inconsistently under field conditions because of competition with native microbiota, soil heterogeneity, environmental variation, and formulation or delivery constraints. Recent microbiome-engineering studies emphasize that successful deployment requires careful design, testing, formulation, ecological compatibility, and field validation rather than simple transfer of laboratory-optimized consortia to agricultural soils [11,70,71].

Overall, plant–microbiome integration strategies represent a promising but still developing strategy for improving root-level N acquisition. Future applications should combine transporter-informed breeding, microbiome management, and fertilization strategies, particularly in systems where organic N mobilization and microbial nutrient cycling strongly influence plant N capture.

## 8. Challenges, Knowledge Gaps, and Future Directions in Transporter-Mediated N Acquisition

### 8.1. Mechanistic Challenges

A central mechanistic challenge is to determine whether transporter-associated microbiome assembly is driven primarily by nutrient-mediated effects, signal-mediated effects, or both. Transporter activity may alter rhizosphere N gradients and substrate availability, whereas root exudates and microbial metabolites may influence microbial recruitment and transporter expression [62]. However, direct causal evidence remains limited and is currently concentrated mainly in rice *NRT1.1B* and OsLHT1 systems [10,11]. Therefore, future studies should distinguish nutrient depletion, altered exudate chemistry, and microbial signal perception as separate but interacting mechanisms.

Another challenge is the limited understanding of the microbial signals that regulate root and transporter activity. Several microbial metabolites, including nitric oxide, phytohormones, volatile compounds, and lipopeptides, may influence root physiology and transporter expression. However, the specific microbial signals, plant receptors and downstream pathways, involved and transporter-mediated and acquisition remained poorly resolved. Identifying these signaling routes will be essential for determining whether transporter microbiome interaction can be predictably manipulated.

### 8.2. Knowledge Gaps

A major knowledge gap is the limited evidence beyond a few transporter genes and crop systems. Current direct evidence is strongest for *NRT1.1B* and *OsLHT1* in rice, whereas the roles of AMTs, AAPs, PTR/POT transporters, and other organic N transporters in microbiome assembly remain largely unexplored [10,11]. This limits the ability to generalize transporter families, -microbiome coupling across crop species, and soil environments.

Another important gap is the limited integration of fungal and mycorrhizal pathways with transporter-mediated N acquisition. Arbuscular mycorrhizal fungi can contribute to plant N uptake and translocation, whereas ectomycorrhizal fungi can mobilize N from organic and mineral-associated pools [14,52,55]. However, these fungal pathways are rarely connected with root transporter genetics or transporter-associated microbiome assembly in crop-focused studies. Incorporating saprotrophic fungi, arbuscular mycorrhizal fungi, and ectomycorrhizal fungi into transporter-microbiome frameworks will be necessary for a more complete understanding of rhizosphere-mediated N acquisition [14,15].

Field validation also remains insufficient. Many transporter and microbiome studies have been conducted under controlled conditions, while rhizosphere interactions in agricultural soils are influenced by soil type, organic matter content, water availability, fertilization regime, native microbial communities, and crop developmental stage. Therefore, field-based studies across contrasting environments are required to determine when transporter-microbiome interactions meaningfully improve root N acquisition.

### 8.3. Future Research Directions

Future research should move from descriptive microbiome profiling toward causal and field-validated tests of transporter–microbiome interactions. Priority questions include: Can targeted editing or selection of N transporter alleles improve recruitment of beneficial rhizosphere microorganisms? Which transporter–microbiome interactions are conserved across rice, wheat, maize, and legumes? How do organic, inorganic, and mixed fertilization regimes alter transporter–microbiome feedback? Can bacterial synthetic communities be combined with fungal or mycorrhizal pathways to improve the stability of N acquisition under field conditions?

Answering these questions will require integrated approaches that combine plant genetics, microbial functional profiling, rhizosphere metabolomics, isotope tracing, and multi-location field trials. Such approaches will help distinguish correlation from causation and identify transporter–microbiome combinations that are robust across soil and management conditions. Ultimately, progress in this field will depend on linking molecular transporter function with microbial N mobilization and practical agronomic performance.

## 9. Conclusions

Root transporters and rhizosphere microbiomes function as interconnected components of belowground N acquisition. Root transporters mediate the uptake of nitrate, ammonium, amino acids, and peptides, while rhizosphere microorganisms regulate the availability of these N forms through organic matter decomposition, mineralization, immobilization, nitrification, fungal decomposition, and mycorrhizal N transfer. Together, these processes create a dynamic soil–root interface in which transporter activity, microbial N mobilization, root exudation, and microbial signaling can jointly influence root-level N capture. Current evidence suggests that specific N transporter genes may contribute to rhizosphere microbiome assembly and transporter-microbiome feedback. However, direct genetic evidence remains limited to a small number of transporter systems and crop species. Therefore, broader validation is required across AMTs, AAPs, PTR/POT transporters, mycorrhiza-associated N transport pathways, diverse crop species, soil types and management regimes. Future integration of transporter-informed breeding, microbiome engineering, and precision fertilization may improve root N acquisition while reducing reliance on synthetic N inputs. However, practical application will require causal evidence, genotype-microbiome compatibility, stable field performance, and careful evaluation across contrasting agricultural environments.

## Data Availability

No new data were created or analyzed in this study. Data sharing is not applicable to this article.
